# Identifying existing management practices in the control of *Striga asiatica* within rice–maize systems in mid‐west Madagascar

**DOI:** 10.1002/ece3.8085

**Published:** 2021-09-12

**Authors:** Donald Scott, Julie Diane Scholes, Meva Tahiry Randrianjafizanaka, Jean Augustin Randriamampianina, Patrice Autfray, Robert P. Freckleton

**Affiliations:** ^1^ Department of Animal & Plant Sciences University of Sheffield Sheffield UK; ^2^ University of Antananarivo Antananarivo Madagascar; ^3^ FOFIFA Antananarivo Madagascar; ^4^ CIRAD Paris France

**Keywords:** cultural control, integrated weed management, legumes, parasitic weeds, sustainable agriculture, witchweeds

## Abstract

Infestations by the parasitic weed genus *Striga* result in significant losses to cereal crop yields across sub‐Saharan Africa. The problem disproportionately affects subsistence farmers who frequently lack access to novel technologies. Effective *Striga* management therefore requires the development of strategies utilizing existing cultural management practices. We report a multiyear, landscape‐scale monitoring project for *Striga asiatica* in the mid‐west of Madagascar, undertaken over 2019–2020 with the aims of examining cultural, climatic, and edaphic factors currently driving abundance and distribution. Long‐distance transects were established across the middle‐west region of Madagascar, over which *S. asiatica* abundance in fields was estimated. Analysis of the data highlights the importance of crop variety and legumes in driving *Striga* density. Moreover, the dataset revealed significant effect of precipitation seasonality, mean temperature, and altitude in determining abundance. A composite management index indicated the effect of a range of cultural practices on changes in *Striga* abundance. The findings support the assertion that single measures are not sufficient for the effective, long‐term management of *Striga*. Furthermore, the composite score has potential as a significant guide of integrated *Striga* management beyond the geographic range of this study.

## Graphical abstract

The parasitic weed genus *Striga* causes huge losses to crop production to subsistence farmers in sub‐Saharan Africa. Here, we report a multiyear, landscape‐scale monitoring project for *Striga asiatica* in the mid‐west of Madagascar. A range of cultural, climatic, and edaphic factors have been identified as driving abundance and distribution, which have significant implications for the management of *Striga*.

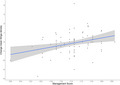

## 1 INTRODUCTION

Species of the genus *Striga*, which belongs to the parasitic plant family Orobanchaceae (Joel et al., 2007), are among the most economically significant weeds affecting food security within sub‐Saharan Africa (SSA) and cause severe losses in many staple crops (Scholes & Press, 2008). *Striga* has resulted in reported yield losses of between 35% and 80% in rice (Rodenburg et al., 2016), 50% and 100% for sorghum (Abunyewa & Padi, 2003), and 21% and 74% for maize (De Groote, 2007). Estimates of economic losses from *Striga* range from between $111 and $300 million per year for rice (Rodenburg et al., 2016) and $383 for maize (Woomer & Savala, 2008). Estimates of the size of the areas affected vary between 50 and 100 million ha annually (FAO, http://www.fao.org/).

Several aspects of *Striga* biology contribute to their invasiveness, persistence, and economic impact. Most significantly, *Striga* species produce exceptionally large numbers of minute seeds (Joel et al., 2013), resulting in the establishment of high population densities over short periods of time (Gressel & Joel, 2013). Seeds can remain dormant within the seed bank for many years, often remaining viable for decades, enabling long‐term persistence in affected areas (Parker, 2013).

In contrast with weed control in high‐intensity agriculture, levels of herbicide use in sub‐Saharan Africa (SSA) remain at very low levels, due to limited access to capital (Grabowski & Jayne, 2016). A recent, comprehensive study of herbicide use within rice production in SSA recorded a mean herbicide frequency of 34% among farmers surveyed (Rodenburg et al., 2019). This study also found low levels of product regulation and frequent suboptimal timing and application techniques. In some SSA countries surveyed, herbicide use was almost nonexistent. For example, in Madagascar only 2% of farmers surveyed used any herbicide (Rodenburg et al., 2019).

Integrated *Striga* management is an initiative that has been promoted by several agencies in different regions of SSA, and uses a combination of approaches to *Striga* control (Baiyegunhi et al., 2019). Integrated *Striga* management incorporates technologies including *Striga* or herbicide‐resistant cultivars (Kanampiu et al., 2003), mycoherbicidal biocontrol (Schaub et al., 2006), arbuscular mycorrhizal inoculates (Lendzemo, 2004), improved tillage, fertilizer inputs (Grenier et al., 2004), and intercrops with legumes (Kamara et al., 2008; Schulz et al., 2003).

In regions where novel technologies promoted by integrated *Striga* management are unavailable, cultural methods to control *Striga* include crop rotation, fallow, and intercropping. Continuous monocropping without rotation leads to increasing levels of infestation and accumulation of *Striga* seed within the soil seed bank (Ejeta, 2007). Increasing the diversity of cropping systems can also contribute to management of conventional weeds while reducing the reliance on chemical inputs, and maintaining crop yields and ecosystem services (Davis et al., 2012). Cultural methods for weed control such as rotation and cultivar selection are well‐established in many agricultural systems in SSA (Rodenburg & Johnson, 2009). Alongside hand weeding, or weeding with hand tools, these are the predominant approaches to weed management in SSA (Lee & Thierfelder, 2017). The use of legumes by intercropping (Bationo & Ntara, 2000), crop rotation, fallow, and agroforestry are also traditionally used to manage soil fertility with respect to N_2_ fixation (Dakora & Keya, 1997).

The incorporation of legumes for cultural management of parasitic weeds in SSA has been documented in a number of studies and shown to be potentially effective. For example, the use of the N_2_‐fixing, woody legume *Sesbania sesban* in fallow in Kenya resulted in seedbank reductions of 50% of *Striga hermonthica* (Oswald et al., 1996). *Cajanus cajan* grown in rotation with maize also resulted in a halving of the density of *Striga asiatica* (Oswald & Ransom, 2001). A study of rice/maize rotations within a no‐till cropping system with permanent soil coverage by a range of legume intercrops found *S. asiatica* infestations were reduced for all rice/maize/legume combinations (Randrianjafizanaka et al., 2018). It is hypothesized that varying rates of N_2_ fixation by different legume crops could influence the ability of a legume crop to control *Striga*. N_2_ fixation alters N availability in soil for host crops. Depletion of soil minerals, including N, has been shown to influence the exudation of root exudates known as strigolactones, which stimulate *Striga* germination and subsequent levels of host infection (Jamil et al., 2011; Yoneyama et al., 2007).

Additionally, legume intercrops can act as “trap” plants and could be important for the reduction in *Striga* seedbanks (Oswald & Ransom, 2001). When intercropped with maize and sorghum, *Glycine max* and *Vigna subterranea* have been shown to cause suicidal germination of *S. hermonthica* seeds, reducing the seedbank (Sauerborn, 1999). This effect has also been observed in *S. asiatica* with intercrops of *Vigna unguiculata* (Ejeta & Butler, 1993).

An further property of intercrops (including legumes) is their ability to shade soils (Carsky et al., 1994). The shading of intercrops can potentially reduce soil temperatures below optimum ranges required for *Striga* germination (e.g., Hsiao et al., 1988; Patterson et al., 1982). Shading by intercrops can also inhibit *Striga* plant development through reduced evapotranspiration rates, which reduce water and nutrient extraction rates from host crops (Stewart & Press, 1990). For instance, field trials using leguminous intercrops of *V. unguiculata* and *G. max* with maize in Kenya recorded suppression of *S*. *hermonthica* germination.

The use of resistant and tolerant crop varieties has also been shown to be an effective method to control *Striga* (e.g., Cissoko et al., 2011; Randrianjafizanaka et al., 2018; Rodenburg et al., 2015). Mechanisms of host resistance to *Striga* can be categorized as either occurring pre‐ or postattachment to the host root system. Preattachment resistance is determined by a reduction in strigolactones, reducing subsequent levels of host infection (Jamil et al., 2011). Strigolactones are signaling compounds, which stimulate the germination of *Striga* (Jamil et al., 2011; Xie et al., 2010). Postattachment resistance refers to the degree in which the haustorium, upon penetrating the host root cortex, then penetrates the endodermis to form a host–parasite xylem connection resistance (Cissoko et al., 2011). In addition, host crop genotypes have been identified which exhibit high degrees of tolerance to *Striga* infection, in terms of photosynthesis, plant height, and yield (Rodenburg et al., 2017).

Field trials are effective in demonstrating the effectiveness of alternative management options at small scales. However, such trials are typically conducted at single sites with limited ranges of variation in environmental conditions. Consequently, there is a question about the effectiveness of various alternatives, when applied in real systems, and across large numbers of farms that vary in terms of soil, history, and management (Freckleton et al., 2018; Rew & Cousins, 2001). In the case of *Striga*, to address this a landscape‐scale study of the drivers of *S. asiatica* distribution was conducted within rice–maize systems in the mid‐west region of Madagascar (Scott et al., 2020). This previous study demonstrated the importance of cultural practices in determining large‐scale distributions of *Striga*, in terms of crop variety, companion crop, and previous crop as well as *Striga* density of the nearest neighboring fields. However, this previous analysis was a static “snapshot” of field densities based on one year's *Striga* density data, without providing information on changes in relation to any management practices. Ideally, tests of the effectiveness of management factors on weed control should use dynamic data that can also account for such temporal variability. Moreover, our previous study did not address the role of several key integrated *Striga* management tools, namely crop rotation and overall crop diversity.

The overall objective of this paper is to test the degree to which existing integrated *Striga* management practices could contribute to the management of *Striga* in the absence of widespread availability of chemical control. Here, we measure the effect of cultural management practices on *Striga* density. These cultural practices include variation in crop variety, intercropping, and use of legumes. In many parts of SSA, this suite of practices represents the main options for cultural weed management. We resurveyed fields over successive years to provide 3 years of crop management data and corresponding changes in weed density between 2019 and 2020.

## 2 METHODS

### 2.1 Study system

Field surveys were undertaken during March 2020, supplementing initial surveys undertaken between February and March 2019. The surveys were undertaken in the mid‐west of Madagascar, one of the six major rice‐growing regions in the country (Fujisaka, 1990). The mid‐west covers 23,500 km^2^, with an elevation between 700 and 1,000 m above sea level. The climate is tropical semihumid, with a warm, rainy season from November to April and a cool, dry season from May to October. Mean annual rainfall ranges from 1,100 to 1,900 mm with a mean temperature of 22℃.

### 2.2 Large‐scale transects

**FIGURE 1:**
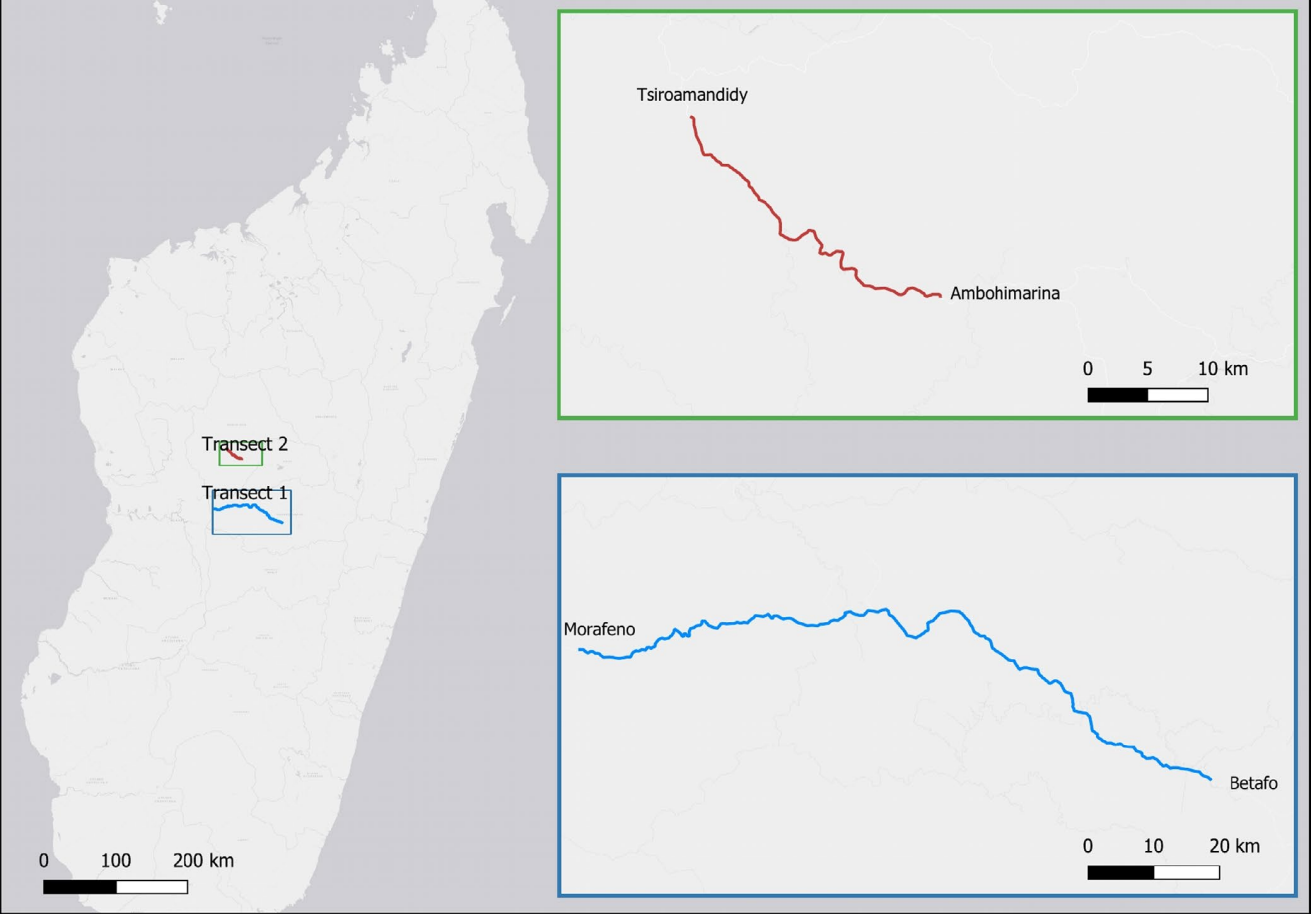
Location of transects 1 and 2, within the Vakinankaratra, Itasy, and Bongolava provinces in the mid‐west of Madagascar

The aim of the sampling was to estimate the abundance of *Striga* within fields that varied in terms of their management. Because access to fields is limited by the absence of good roads, we structured our survey program around the main road system. Field sampling was based around two long‐distance‐driven transects along which *Striga* abundance was estimated in fields adjacent to the road. These comprised a transect of 129 km along the RN34 and one of 25 km along the RN1b. A total of 221 fields were surveyed (transect 1: *n* = 174, transect 2, *n* = 47). Transect 1 was located within Vakinankaratra province, between the towns of Betafo and Morafeno, and transect 2 was located within Itasy and Bongolava provinces, approximately 6 km east of Ambohimarina and the outskirts of Tsiroanomandidy (Figure 1). Rice–maize cropping systems are largely employed within the study areas, with incorporation of legumes—mainly cowpea (*V. unguiculata*), ricebean (*Vigna umbellata*), soybean (*G. max*), and groundnut (*Arachis hypogaea*)—and manioc (*Manihot esculenta*).

Fieldwork was undertaken with support from local technicians and guides who were familiar with the locality and field history. Prior to commencing work within a locality, the Chef Fokotany (local administrative head) was sought in order to present ourselves and detail the work we were undertaking.

### 2.3 Within‐field sampling

One field was surveyed on adjacent sides of the road every kilometer. During the initial surveys in 2019, it was quickly established that detection of *S. asiatica* was possible within pluvial rice and maize fields of typically planted densities at distances up to 5 m on either side of each surveyor. Quadrat dimensions of 200 m^2^ (10 × 20 m) were agreed based on a trade‐off between speed of data capture and accuracy of measurement. Fields were divided into pairs of 10 × 20 m quadrats, in which two observers simultaneously recorded *Striga* density, by walking at a steady pace along a central transect, and scanning 5 m to either side; in fields >1,200 m^2^, data were recorded from a maximum of three pairs of quadrats. A field corner was randomly selected as the starting point for each field survey. *Striga* density was estimated using a six‐point, density‐structured scale, ranging from absent (0) to very high (5). Definitions of density states were determined during fieldwork in 2019, and a table with narrative descriptors of the scale used alongside representative photographs for each density state was produced (see Appendix S1).

Information was collated on crop type, rice variety, companion crop, and previous crop. In addition, mean crop height and percentage crop cover were estimated for each quadrat. Mean density score for *Striga*, average crop height and cover, and other weed cover for a quadrat were entered on a mobile application prior to moving to a subsequent quadrat. If no *Striga* was found in a quadrat, a thorough walk throughout the entire field was undertaken to verify that *Striga* was truly absent. If *Striga* was then located, density was estimated for this area which replaced a quadrat with a zero record on the data sheet.

To measure changes in *Striga* density between years, fields surveyed in the first year (2019) were relocated using a GPS‐enabled smartphone. Data were recorded using a smartphone with the mobile application “Google Sheets” (Google LLC, 2020, version 1.20.492.01.45) to allow rapid and paperless data entry. Where new fields were surveyed, georeferencing was undertaken using “Google My Maps” (Google LLC, 2020, version 2.2.1.4).

In a small number of instances, it was not possible to verify the exact location of previously surveyed fields. This was a consequence of GPS error, resulting in coordinates being located in margins between small fields, or being clearly erroneous (e.g., centered on a road, nonagricultural location). In these instances, the field was omitted (*n* = 19). In instances where the resurveyed field contained a current nonhost (i.e., noncereal) crop, the field was surveyed but was omitted from analyses of *Striga* density (*n* = 55). An adjacent, substitute field containing a cereal crop was surveyed and added to the dataset. Of the resurveyed noncereal crop fields, only three were found to contain low, residual levels of *Striga*.

Our initial intention was to extend both transects in order to capture a greater degree of altitudinal and climatic heterogeneity. However, owing to logistic constraints imposed by the COVID‐19 situation, it was only possible to extend transect 1 by 16 kilometers east. It was also not possible to either resurvey the entirety of fields originally surveyed in 2019 or extend transect 2.

### 2.4 Soil samples

Alongside the impact of cropping, the role of available nitrogen in determining *Striga* densities was addressed through collecting and analyzing soil samples for NO_3_. These samples were collected in pairs from quadrats with contrasting *Striga* densities within the same field. Samples comprised 23 pairs representing differing densities from absent to very high. These were analyzed immediately following collection, with data added to those of the 98 samples collected in 2019 for the purposes of analysis.

Soil samples were obtained from the center of each selected quadrat using a 20 mm diameter, hand‐held, tubular soil sampler to a depth of approximately 20 cm. Soil samples were subsequently air‐dried for analysis.

NO_3_ analysis was undertaken using a LAQUAtwin NO_3_‐11 nitrate meter (Horiba Scientific). Owing to low levels of NO_3_ within the soil, it was necessary to dilute the standard solution supplied with the meter. Therefore, calibration was undertaken between 15 and 150 ppm NO_3_ to improve sensitivity. One gram of dried soil was mixed with one milliliter of water and ground in a pestle and mortar. The resultant solution was then placed on the sensor of the meter. This procedure was repeated a minimum of two times per soil sample. If agreement between the first two readings was observed (i.e., between ±5 ppm NO_3_ between readings), then the readings were taken, and the mean of the readings was used. If the readings did not concur, then sampling was repeated until stabilization of readings.

### 2.5 Climate and altitude

Climate data were obtained from the WorldClim2 dataset (Fick & Hijmans, 2017). Climatic parameters included in the analyses were mean annual rainfall and mean annual temperature. Precipitation seasonality was included as an additional climatic factor. This was obtained by calculating the coefficient of variation (CV) of mean monthly precipitation, which is the ratio of the standard deviation of the monthly total precipitation to the mean annual precipitation (O'Donnell & Ignizio, 2012). Invasion risk modeling has identified the seasonality of precipitation as one of the most significant bioclimatic variables influencing the occurrence of *S. asiatica* (Mudereri et al., 2020). Seasonality is the chief driver of variation in monthly rainfall through the year. Therefore, the CV of monthly precipitation is an appropriate measure of seasonal variation. Altitudes for surveyed sites were obtained from CGIAR—Consortium for Spatial Information (CGIAR‐CSI, 2019).

### 2.6 Statistical methods

Linear models were used to test the effects of management (rice variety, previous crop, and companion crop) and climatic predictors (mean annual temperature, mean annual rainfall, altitude). Soil sample data from 2019 and 2020 were analyzed, using NO_3_ as a predictor of *Striga* density. Within‐field *Striga* density was also plotted against that of neighboring fields. Year was also included in interaction with all predictors in order to test for any differences in patterns between the 2 years.

*Striga* density was log (*x* + 1)‐transformed owing to the presence of large numbers of zero densities. Categorical variables incorporated into the models included crop variety, previous crop, and companion crop. We included and tested terms sequentially (using type I sums of squares): Specifically, the interaction between year and the main effects was included and tested as the final variable in the model to maintain the principle of marginality.

Statistics were calculated using R 3.6.3 (R Core Team, 2020) and the packages: dplyr (v0.8.0.1; Wickham et al., 2015), mgcv (Wood, 2011), lme4 (v067.i01, Bates et al., 2015), lmerTest (Kuznetsova et al., 2017), MASS (Venables & Ripley, 2002), DescTools (v 0.99.28, Signorell et al., 2019), and psych (Revelle, 2018, v1.8.12). The full reproducible code is available in Appendix S2.

Tests for collinearity between climatic factors indicated strong correlation between mean temperature and precipitation seasonality (*f* = 1,768.9, *df* = 1, 406, *R* ^2^ = 0.81, *p* < 2.2e−16, VIF: 5.36, see plot, Appendix S3). This is because higher temperatures are correlated with greater variation in seasonal rainfall. Owing to this correlation, these terms were included in separate models.

### 2.7 Legume crops

We tested the effects of the incorporation of legumes into crop rotation, as well as to examine effects of individual legume crops on *Striga* density. This analysis used data from all fields surveyed in 2019–2020, in which either a current legume companion crop or a previous legume crop was recorded. Firstly, a linear model was used to determine binary effects of the presence or absence of legumes in rotation using log‐transformed mean *Striga* density. Secondly, an analysis was undertaken to examine the effects of individual legume crops on *Striga* density using mean *Striga* density (log‐transformed) as the response for a linear model.

### 2.8 Management and change in *Striga* density

In the set of analyses described above, the objective is to determine which factors correlate with the density of *Striga*. However, this does not tell us whether the correlates of static density measures are able to predict the impact of management on the *change* in density from one year to the next. Therefore, we tested whether models fitted to static density data could predict changes in *Striga* density.

Based on the outcome of the models described above, we tested the combined effects of a suite of management factors thought to individually affect *Striga* density, specifically inclusion of fallow, number of years of cereal cultivation, number of years of legume crop cultivation, and crop diversity (see Table 1). This analysis used cropping data obtained from field survey combined across 2019 and 2020 and included the previous crop for 2019, therefore, giving a three‐year sequence of crop rotation data.

**TABLE 1:** Management scores for individual practices, calculated from verifiable 2‐year dataset including previous crop for 2019

Variable	Range	Coefficient
Fallow	0–1	−0.2018*n*
Years of cereal planting	2–3	−0.09133*n*
Years of legume planting	0–3	−0.36512*n*
Crop diversity	1–5	−0.26289*n*

Note

These measures were scaled using coefficients derived from a linear model including all four factors and summed to produce an overall *Striga* “management score” for each field.

We fitted a single linear model using the four individual factors (fallow, years of cereal cultivation, years of legume crop cultivation, and crop diversity) as predictors of *Striga* density. The resultant values were then summed to produce a composite score (Table 1). Example calculations for fields with different indicator scores are provided in Table 2. The composite scores were then used as the independent variable in a linear model of change in mean *Striga* density between 2019 and 2020 as the response.

**TABLE 2:** Example calculations for fields with differing composite scores

FL_YR	Score	CR_YR	Score	LM_YR	Score	NC	Score	Total
No	0	2	−0.18266	1	−0.36512	3	−0.78867	−1.33645
Yes	−0.2018	2	−0.18266	2	−0.73024	4	−1.05156	−2.16626
No	0	2	0.18266	2	−0.73024	4	−1.05156	−2.16626
Yes	−0.2018	2	0.18266	1	−0.36512	4	−1.05156	−1.80114
No	0	3	−0.27399	0	0	2	−0.52578	−0.79977

Note

FL_YR = Fallow included in 3‐year rotation, CR_YR = Number of years of cereal planting in 3‐year rotation, LM_YR = Number of years in which legumes have been planted in 3‐year rotation, NC = Number of different crops planted in 3‐year rotation.

The score for legume crops included fields containing *Mimosa diplotricha*. Though this appeared to be an incidental weed species, its properties as an N‐enriching green manure species are well‐established (Thomas & George, 1990; Yogaratnam et al., 1984). To simplify, and based on the results of models fitted to statistic density data, no differentiation was made between legume species. However, different rice varieties were considered as separate crops, owing to their observed influence on *Striga* density (Cissoko et al., 2011; Randrianjafizanaka et al., 2018; Rodenburg et al., 2015; Scott et al., 2020).

## 3 RESULTS

**FIGURE 2:**
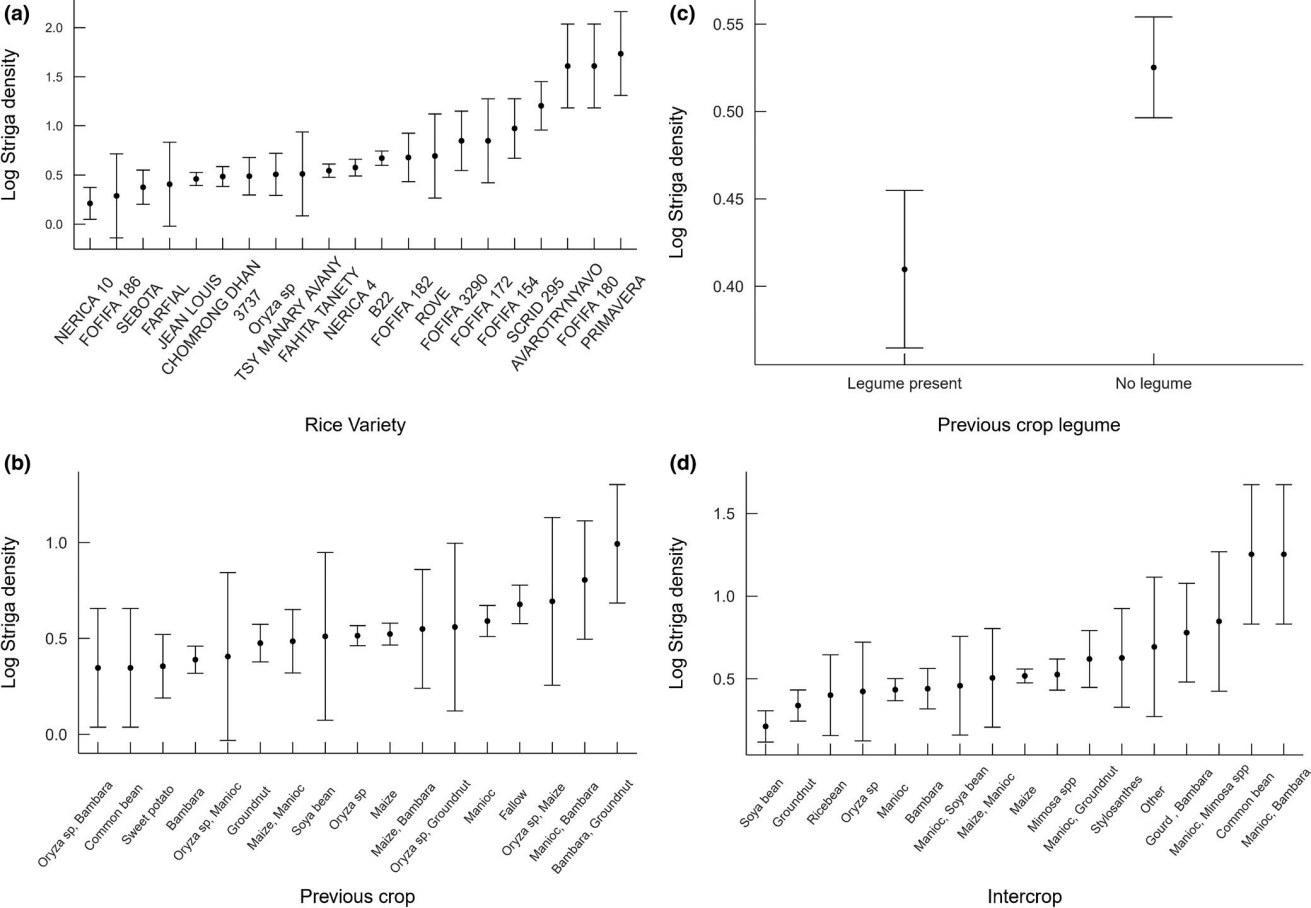
(a) Log *Striga* density for rice variety ± SE, NERICA‐10 *n* = 10, FOFIFA 186 *n* = 1, SEBOTA *n* = 6, FARFIAL *n* = 1, JEAN LOUIS *n* = 44, CHOMRONG DHAN *n* = 18, 3,737 *n* = 5, *Oryza* sp. *n* = 4, TSY MANARY AVANY *n* = 1, FAHITA TANETY *n* = 43, NERICA‐4 *n* = 33, B22 *n* = 41, FOFIFA 182 *n* = 4, ROVE *n* = 1, FOFIFA 3290 *n* = 2, FOFIFA 172 *n* = 1, FOFIFA 154 *n* = 2, SCRID 295 *n* = 3, AVAROTRYNYAVO *n* = 1, PRIMAVERA *n* = 1), (b) Log *Striga* density for previous crop ± SE, (onion *n* = 1, *Oryza* sp./Bambara groundnut *n* = 2, common bean *n* = 2, sweet potato *n* = 7, *Oryza* sp./manioc *n* = 1, groundnut *n* = 20, maize/manioc *n* = 7, soybean, *n* = 20, *Oryza* sp. *n* = 70, maize *n* = 59, maize/Bambara groundnut *n* = 2, *Oryza* sp./groundnut *n* = 1, manioc *n* = 29, fallow *n* = 19, *Oryza* sp./maize *n* = 1, manioc/Bambara groundnut *n* = 2, Bambara groundnut/groundnut *n* = 2. (c) Log *Striga* density for previous crop type ± SE (‐legume/nonlegume). (d) Mean *Striga* density for companion crop ± SE (soybean *n* = 20, groundnut *n* = 20, ricebean *n* = 4, *Oryza* sp. *n* = 2, manioc *n* = 40, Bambara groundnut *n* = 13, manioc/soybean *n* = 2, maize/manioc *n* = 2, maize *n* = 101, *Mimosa* spp. *n* = 20, manioc/groundnut *n* = 6, Stylosanthes *n* = 2, other *n* = 2, gourd/Bambara groundnut *n* = 2, manioc/*Mimosa* spp. *n* = 1, common bean *n* = 1, manioc/Bambara groundnut *n* = 1 ± SE

The importance of the rice variety and whether the previous crop was leguminous were evident in this dataset (Table 3, Figure 2). Rice variety NERICA‐10 was associated with lowest mean *Striga* densities (see also Scott et al., 2020). Several locally improved varieties (FOFIFA/SCRiD) and landraces are associated with higher *Striga* densities.

**TABLE 3:** Summary of models relating density of *Striga* to a range of management and ecological predictors

Variable	Year	(*df*)	*p*	Effect	(*df*)	*p*	Year × effect	(*df*)	*p*
(a) Management variables									
Rice variety	0.57	(1, 164)	.450	2.02	(27, 164)	**.004**	1.90	(9, 164)	**.054**
Previous crop	3.25	(1, 238)	.073	1.02	(23, 238)	.434	2.21	(6, 238)	**.043**
Companion crop	11.52	(1, 209)	**.001**	1.13	(25, 209)	.315	0.48	(6, 209)	.822
Previous legume	4.33	(1, 316)	**.038**	6.39	(1, 316)	**.012**	0.02	(1, 316)	.885
Legume crop	8.69	(1, 133)	**.004**	1.82	(6, 133)	.099	2.37	(3, 133)	.073
(b) Ecological variables									
Neighbor density	3.04	(1, 338)	.082	5.83	(1, 338)	**.016**	6.32	(1, 338)	**.012**
Mean rainfall	5.94	(1, 411)	**.015**	1.84	(1, 411)	.162	14.29	(1, 411)	**.000**
Precipitation seasonality	5.87	(1, 411)	**.016**	8.78	(1, 411)	**.003**	3.13	(1, 411)	.078
Altitude	5.56	(1, 409)	**.019**	9.20	(1, 409)	**.003**	0.51	(1, 409)	.478
Mean temperature	5.89	(1, 411)	**.016**	12.61	(1, 411)	**4.3** × **10^–4^**	0.58	(1, 411)	.448
NO3	0.293	(1, 69)	.590	0.10	(1, 69)	.752	0.19	(1, 69)	.663
Other weed cover	5.69	(1, 337)	**.018**	1.46	(1, 337)	.227	0.10	(1, 337)	.750

Note

Precipitation seasonality (coefficient of variation for rainfall) is included as an additional test for the combined dataset. Resurvey in 2020 included a subset of original fields with additional fields. Updated analyses used combined dataset for both years.

**FIGURE 3:**
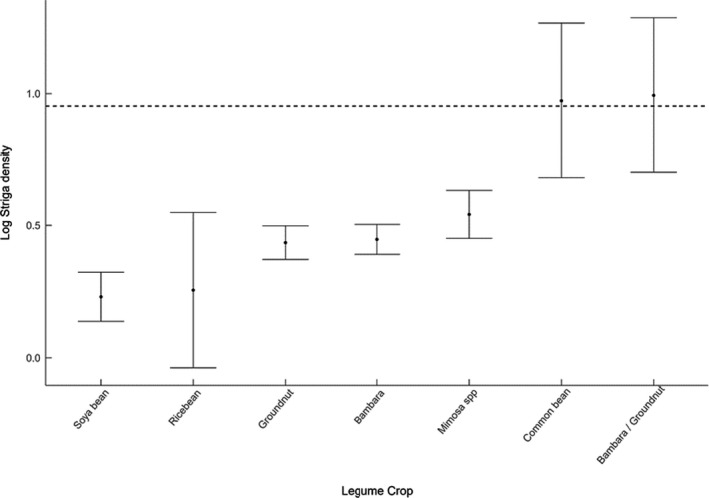
Log *Striga* density for fields planted with either a current legume companion crop or previous legume crop ± SE and grand mean (dashed line), soybean *n* = 20, AH, VU (*Vigna umbellata*) *n* = 2, groundnut *n* = 42, Bambara groundnut *n* = 54, *Mimosa* spp. *n* = 21, common bean *n* = 2, Bambara groundnut/groundnut *n* = 2

Fields previously planted with legumes had significantly lower densities than those that had not (Table 3 & Figure 2c). The linear model using individual legume crops as the independent variable for *Striga* density did not indicate any significance for this factor, with the majority of variation explained by the effect of year. However, Figure 3 indicates varying levels of *Striga* infestation associated with different legume crops.

### 3.1 Impacts of management diversity

Patterns of rotation of main crops between years are shown in Table 4. Crop rotations were dominated by cereal (rice/maize) accounting for 44.5% of all combinations, comprising continuous maize (15%), continuous rice (10%), and followed by maize/rice or rice/maize (19%). Following this was rice/maize and Bambara groundnut (13%), rice/maize and manioc (10%), rice/maize and groundnut (7%), and rice/maize and fallow (6%). Soya and other legumes were less widely recorded as a main crop, but were more frequently recorded as an intercrop.

**TABLE 4:** A transition matrix illustrating rotations for main crops recorded for the study between 2020/2019 and previous main crops recorded in fields for 2019

	First crop								
	Rice	Maize	Fallow	Manioc	Bambara*	Cowpea*	Groundnut*	Soya*	Sweet potato
Second crop									
Rice	29	37	8	9	5	0	0	2	1
Maize	16	43	2	0	0	0	0	0	0
Bambara*	28	9	0	1	0	0	0	0	0
Manioc	16	13	0	1	0	1	0	0	0
Fallow	12	6	0	0	1	0	0	0	0
Common bean*	1	1	0	0	0	0	0	0	0
Groundnut*	9	11	0	0	0	0	0	0	0
Maize/Manioc	2	5	0	0	0	0	0	0	0
Manioc/Bambara*	1	1	0	0	0	0	0	0	0
Rice/Bambara*	2	0	0	0	0	0	0	0	0
Soya*	1	0	0	0	0	0	0	0	0
Sweet potato	3	4	0	0	0	0	0	0	0

Note

The number in each cell is the number of fields for each rotation. The color represents the number of fields in each observed rotation. Asterisk denotes legume crop.

**FIGURE 4:**
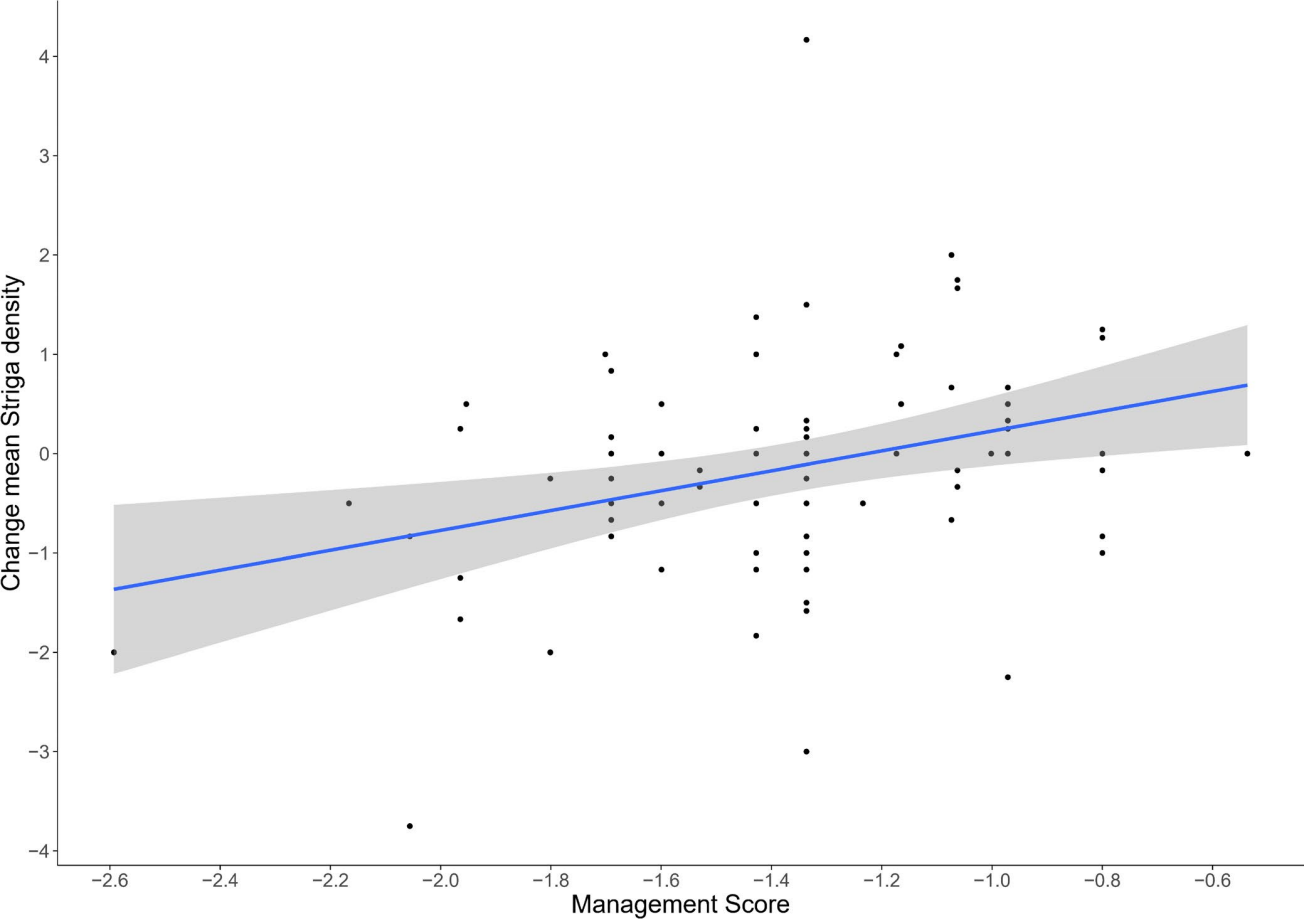
Change in mean *Striga* density and composite management score. Score comprised: years of fallow, number of years of cereal cropping, number of years of legume cropping, and number of different crops planted. Values were weighted using coefficients derived from a linear model containing each factor as individual terms. As all coefficients were negative, a higher score is associated with increases in *Striga* density. The effect of management score on change in mean *Striga* density was significant for both the linear model

**FIGURE 5:**
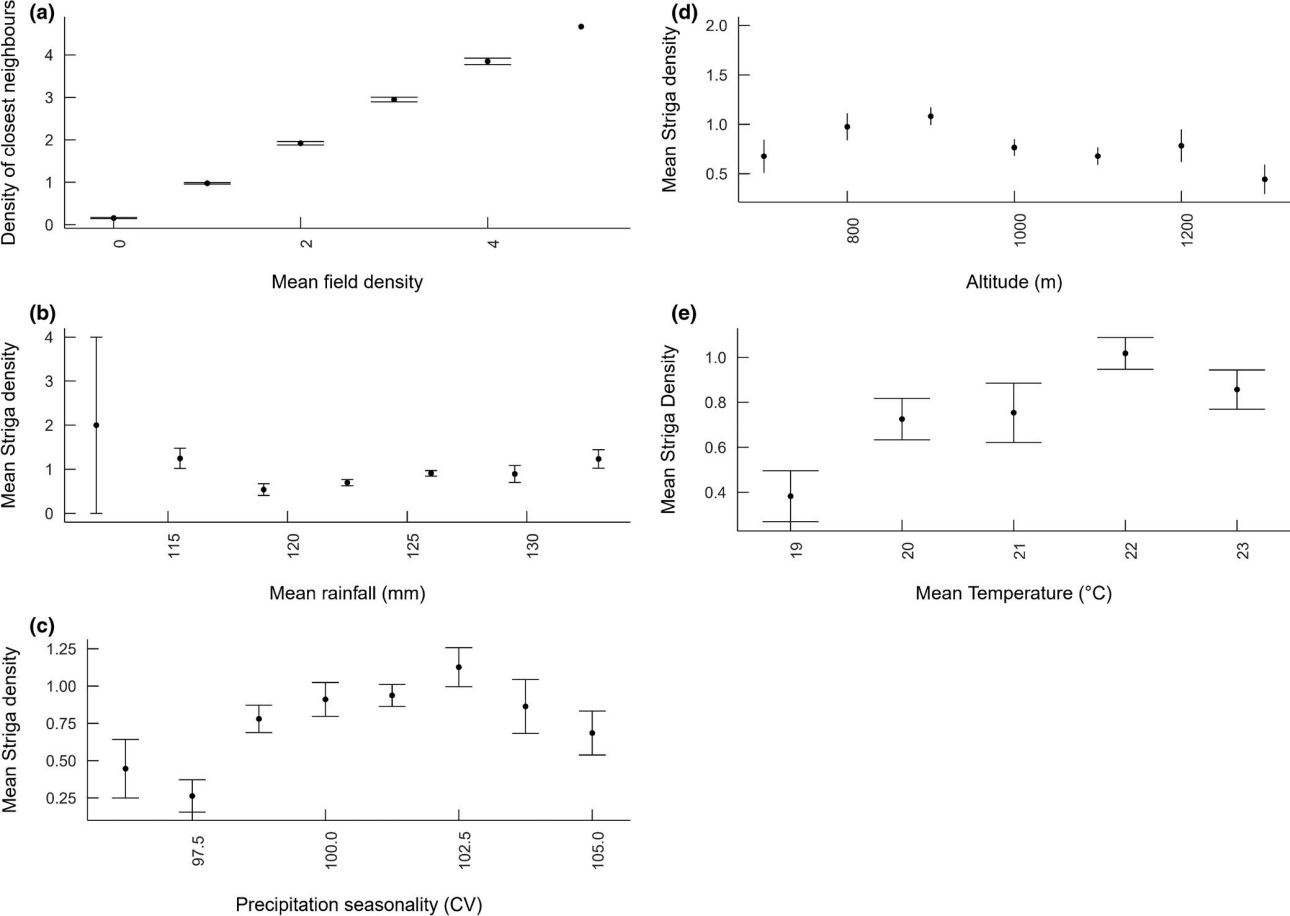
(a) Mean within‐field *Striga* density and *Striga* density within closest neighboring fields ± SE, (b) mean *Striga* density and mean annual rainfall ± SE, (c) mean *Striga* density and precipitation seasonality (coefficient of variation for rainfall) ± SE, (d) mean *Striga* density and altitude ± SE, (e) mean *Striga* density and mean annual temperature ± SE. The effects of both neighboring densities, precipitation seasonality, altitude, and mean temperature on mean *Striga* density were significant for linear models (see Table 3)

Results for the analyses of the composite management score indicated significant effects on change in *Striga* density (*F* = 9.06, *df* = 1, 76, *p* = .0035). Figure 4 indicates a clear positive relationship between the composite of management index scores for fields and mean change in *Striga* density between 2019 and 2020. The strong effect of *Striga* abundance of neighboring fields suggests that this is a very strong predictor of within‐field density (see Figure 5a, Table 3). This reinforces previous results (Scott et al., 2020) and suggests that spatial factors are important in determining *Striga* distribution and spread.

Significant effects for precipitation seasonality, altitude, and temperature were indicated as follows: with distinct trends in density observable along individual gradients (Figure 5c–e). Soil analyses produced similar results with no significance of probabilities, in line with analysis of 2019 data alone.

Year emerged as significant term in the majority of models (companion crop, previous crop legume, legume crop, mean rainfall, precipitation seasonality, altitude and mean temperature, and other weed density) and as an interaction term in models for rice variety, previous crop, neighboring density, and mean rainfall (Table 3).

## 4 DISCUSSION

This study provides evidence of the effect of a wide range of individual factors on *Striga* abundance at a landscape scale over multiple years. Given the importance of rice variety, legume crops, and *Striga* density within adjacent fields, we provide evidence to contribute to the multifactor approach to *Striga* through integrated *Striga* management. The identification of year as a consistently significant effect across models illustrates the importance of interannual variability of *Striga* density. Strong interannual variation in *Striga* density has also been observed by other multiyear studies of cropping practices on *Striga* density (Khan et al., 2007; Midega et al., 2014; Randrianjafizanaka et al., 2018; Reda et al., 2005).

The work presented here advances our previous work in several respects. Firstly, the expansion of ranges encompassed by the 2020 surveys showed the significance of climatic and altitudinal factors in determining *Striga* density, not revealed in the analysis of the 2019 alone. Secondly, recording interannual variability in *Striga* density allowed for the assessment of the effects of a number of combined cultural factors. This is significant from a management perspective as it provides evidence of measures which can be implemented to control this problematic weed.

### 4.1 Climate and altitude

The significant effect of precipitation, seasonality, and mean temperature in our data concurs with ecological niche modeling, field surveys, and laboratory tests undertaken elsewhere. Mudereri et al. (2020) used a range of models including bioclimatic variables to determine the ecological nice of *S. asiatica* in Zimbabwe. Precipitation seasonality was consistently identified as a key factor within all models. Niche‐based modeling prediction undertaken by Mandumbu et al. (2017) also identified precipitation variation as a major determinant of future spread. An association between regions with erratic, savannah‐type rainfall patterns, and high rates of *Striga* infestation has also been noted from field surveys (Dugje et al. (2006). The role of moisture variation in *Striga* seed conditioning and germination has also been demonstrated in laboratory studies (e.g., Babikar et al., 1987; Hsiao et al., 1987; Mohamed et al., 1998).

A minimum seed conditioning and germination temperature of 20℃ for *S. asiatica* was observed by Hsiao et al. (1988) and Patterson et al. (1982). Patterson (1990) suggested that *S. asiatica* requires a mean temperature of 22℃ to reach maturity, with an optimum temperature of 32℃. While there are a few observations from this study, which fall below these thresholds; the general trend supports the assertion of these temperature ranges.

The significance of altitude as a predictor of *Striga* density is evident. Figure 5d shows fields with highest infestation rates occurring at intermediate altitudes. Rodenburg et al. (2014) also observe that *S. asiatica* is particularly problematic at altitudes between 800 and 1,100 m a.s.l within the region of Vakinankaratra, which serves to confirm this observation.

### 4.2 Soil NO_3_

*Striga* density was not found to be related to NO_3_ levels in the soil. There are several potential reasons for this. Firstly, the literature suggests contradictory effects of the role of nitrogen on *Striga* emergence. For example, although Osman et al. (1991) recorded a significant increase in emerged *S. asiatica* between plots with applied nitrogen versus nitrogen‐poor controls, no significance was found in numbers of emerged *Striga* between N treatments. However, Mumera and Below (1993) found decreases in *S. hermonthica* with increased rates of applied N, although interannual variability was considerable.

A second factor in the lack of observed impact of NO_3_ is the timing of sampling. NO_3_ samples were collected just before harvest at the end of the growing season. Soil N rates in rainfed rice are highest at the time of crop planting, with plant uptake and leaching decreasing over the duration of the growing season (Ranaivoson et al., 2019). Timing of sampling is therefore a possible factor in the lack of recorded effects of NO_3_ on *Striga* density.

### 4.3 Legumes

The results of this study demonstrate the effect of legumes cropping systems on *Striga* density on a number of levels. Firstly, the effect of legumes in general was demonstrated by the lower mean *Striga* density associated with the previous planting of legumes versus other crop types (Figure 2c). The generalized effect of legumes was further supported by the significance of the composite management score, which includes number of legumes planted over a three‐year rotation as a component (Figure 4). Although individual legume crops show varying mean *Striga* densities in Figure 4, these differences were not significant, with significance within this model apportioned to year.

The individual effects of legume crops on *Striga* density also vary between other comparable studies. For example, Randrianjafizanaka et al. (2018) recorded significant effects of a cowpea, Mucuna, ricebean, and *Stylosanthes* intercrops on *S. asiatica* density in both rice and maize. A study by Khan et al. (2007), using common bean, cowpea, *Crotalaria*, *Desmodium*, mung bean, and groundnut, only found a significant effect for *Desmodium* intercrop. Midega et al. (2014) only found significant differences among some legumes in certain cropping seasons, while Reda et al. (2005) found no significance for a suite of legume intercrops.

### 4.4 Management

The analysis of the management score indicates a significant relationship between the combined factors and interannual variation in *Striga* density. While these variables when assessed individually may not demonstrate significant effects due to their coarse resolutions, their combined effect on change in *Striga* density is considerable from a farm management viewpoint. Indeed, the importance of an integrated *Striga* management approach, combining multiple methods has been demonstrated in several other studies (e.g., Randrianjafizanaka et al., 2018; Tesso & Ejeta, 2011).

Effective dissemination of novel technologies associated with integrated *Striga* management requires functional and accessible extension services to maximize farmer's awareness and education (Ellis‐Jones et al., 2004; Emmanuel et al., 2016). Increased costs associated with implementing novel‐integrated *Striga* management technologies are also related to adoption rates, with larger commercial farmers showing significantly higher levels of adoption in other areas of SSA (Baiyegunhi et al., 2019). Both these factors represent significant barriers to both diffusion and adoption of new integrated *Striga* management technologies in Madagascar.

Extension services are not sufficient to effectively support widespread diffusion of other novel technologies (Harvey et al., 2014). In addition, around 70% of farmers in Madagascar practice subsistence agriculture (Institut National de la Statistique de Madagascar (INSTAT), 2010, 2011), while the average farm area for upland rice for Madagascar is 1.28 ha (Zeller et al., 1999). Agriculture is also subject to frequent extreme weather events and pest and disease infestations (Rakotobe et al., 2016). Coupled to this is an absence of financial safety nets and widespread food insecurity for at least part of the year (Harvey et al., 2014). These factors result in an understandably high degree of risk‐aversion toward adopting new technologies, even when they are available (Moser & Barrett, 2003). Therefore, the adaption of existing practices, combined with available resistant crops, is considered a more viable approach to *Striga* management within this context.

Because of the complexity of the information included, we simplified by developing a management score designed to represent the complexity and diversity of crops used. The use of composite indices is an effective means of aggregating often‐disparate individual indicators into a single summary value (Foster et al., 2013; Greco et al., 2019). Such indices have the potential to summarize systems in ways not directly measurable (Dobbie & Dail, 2013). They have been widely used within ecological and environmental assessments, for example, to measure biotic integrity of freshwater and riparian habitats (Karr, 1981; Munné et al., 2003), assess habitat suitability for protected species (Oldham et al., 2000), and measure global biodiversity trends (Collen et al., 2009) and national‐level environmental performance (Srebotnjak, 2014)).

## 5 CONCLUSION

The findings of this study further demonstrate the influence of a range of individual cultural factors on *Striga*. Moreover, the influence of individual legume crops on *Striga* density provides additional insight into observations of overall effects of legumes in general. Further study of the degree to which these effects are attributable to either the habit or N fixing properties of different legume crops is recommended to obtain a deeper understanding of the specific roles of different legume crops.

The analysis shows, however, that no single factor influences *Striga* density to the degree that it can be considered a panacea for control. Indeed, it is widely accepted that single measures are not sufficient for the effective, long‐term management of *Striga*. The influence of the composite management score in reducing *Striga* densities is of potential relevance to farmers and extension workers in regions without access to novel control technologies. The scoring system provides an indication of the way in which several, easily measurable factors combine to result in significant reductions in *Striga* density between years. With annual monitoring, the index could be employed as an adaptive management tool, providing feedback on changes in infestation and options to adapt cropping accordingly. If used as a complementary method, alongside locally effective resistant crop varieties and legume intercrops, the composite score has potential as a significant component of integrated *Striga* management beyond the geographic range of this study.

## CONFLICT OF INTEREST

The authors have no conflicts of interest to declare.

## AUTHOR CONTRIBUTIONS

**Donald Scott:** Conceptualization (equal); Data curation (lead); Formal analysis (equal); Investigation (lead); Methodology (equal); Project administration (lead); Resources (lead); Validation (equal); Visualization (lead); Writing—original draft (lead); and Writing—review & editing (equal). **Julie Diane Scholes:** Conceptualization (supporting); Funding acquisition (equal); Investigation (supporting); Methodology (supporting); Project administration (supporting); Resources (supporting); Supervision (supporting); Validation (supporting); and Writing—review & editing (supporting). **Meva Tahiry Randrianjafizanaka:** Investigation (supporting); Methodology (supporting); and Writing—review & editing (supporting). **Jean Augustin Randriamampianina:** Investigation (supporting); Methodology (supporting); and Writing—review & editing (supporting). **Patrice Autfray:** Conceptualization (supporting); Formal analysis (supporting); Investigation (supporting); Methodology (supporting); Project administration (supporting); Resources (supporting); Supervision (supporting); Validation (supporting); and Writing—review & editing (supporting). **Robert P. Freckleton:** Conceptualization (equal); Data curation (supporting); Formal analysis (equal); Funding acquisition (lead); Investigation (supporting); Methodology (equal); Project administration (supporting); Resources (equal); Supervision (lead); Validation (lead); Visualization (equal); Writing—original draft (supporting); and Writing—review & editing (equal).

## Supporting information

Appendix S1

Appendix S2

Appendix S3

## Data Availability

The datasets and code generated during and/or analyzed during the current study are available in the Dryad repository: (https://doi.org/10.5061/dryad.4qrfj6qb3).
