# Supplementary material for: Identifying existing management practices in the control of Striga asiatica within rice–maize systems in mid‐west Madagascar
Source: Ecol Evol. 2021 Sep 12;11(19):13579–92. doi: 10.1002/ece3.8085 (PMC8495792; doi:10.1002/ece3.8085)
Supplement: Supplementary file 1 — Appendix S1 [file ECE3-11-13579-s003.docx]

| Density | Description |
| --- | --- |
| 0 (Absent) | No *Striga* present either within quadrat or within field (dependent on scale of determination). In case of field scale, extensive search undertaken across entire field to determine absence. |
| 1 (Very Low) | Between one and ten percent of host crop plants infected recorded across the quadrat. |
| 2 (Low) | Between 11 and 25 percent of host crop infected across the quadrat. Crop symptoms unlikely to be easily visible / or attributable to *Striga.* |
| 3 (Moderate) | Between 26 and 50 percent of host crop plants infected across the quadrat. Localised visible stunting, chlorosis, wilting and poor yield most likely attributable to *Striga* damage. |
| 4 (High) | Between 51 and 75 percent of host crop infected across the quadrat. Widespread visible stunting, chlorosis, wilting and visibly poor yield across majority of host crop, directly attributable to *Striga.* |
| 5 (Very High) | Between 76 and 100 percent of host crop plants infected across the quadrat. Stunting, chlorosis and wilting resulting in almost or complete crop failure. |

# Appendix 1 Striga Density state guides

Appendix 1a: Descriptions of Striga asiatica density states from absent to very high Striga infestation.


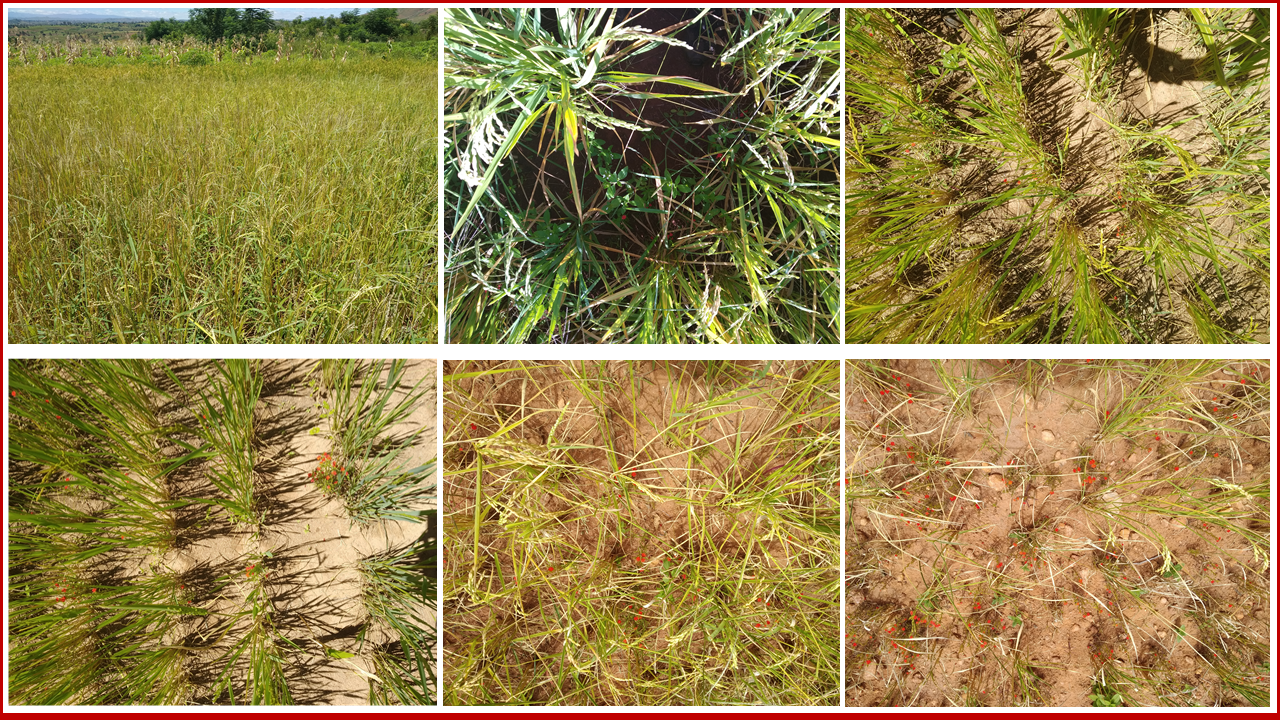


Appendix 1 b: Indicative photographs for rice field in each estimated state from absent (top left) to very high Striga infestation (bottom right).
