# Supplementary material for: Identifying existing management practices in the control of Striga asiatica within rice–maize systems in mid‐west Madagascar
Source: Ecol Evol. 2021 Sep 12;11(19):13579–92. doi: 10.1002/ece3.8085 (PMC8495792; doi:10.1002/ece3.8085)
Supplement: Supplementary file 2 — Appendix S2 [file ECE3-11-13579-s001.docx]

# Appendix 1: Model details, outputs and R scripts

| **Model** | **#** | **Code** | **Result** |
| --- | --- | --- | --- |
| Log Striga density V Year * NO3 | LM1 | library(mgcv)  library(lme4)  library(lmerTest)  library(ggplot2)  library(dplyr)  library( geosphere )  library( stringr)  #Calculate a standard error  stderr <- function(x, ...) sd(x, na.rm = TRUE) / sqrt(length(is.na(x == FALSE)) )  # Have cleaned the cultivar variable  fulldata <- read.csv("/Users/Ragenaky/Desktop/Striga Madagascar 2020/Completed Sheets/Ecology Data Updated/MASTER_2019_2020_NANC.csv", h = T)  fulldata$YR <- as.factor(fulldata$YR)  fulldata$FN <- as.factor(fulldata$FN)  # Load NO3 data  NO3 <- read.csv("/Users/Ragenaky/Desktop/Striga Madagascar 2020/Completed Sheets/Ecology Data Updated/NO3_2019_2020.csv", h = T)  colnames(NO3)[1] <- "YR"  NO3Summary <- NO3 %>%  group_by(YR, Field) %>%  summarise( NO3 = mean(NO3_ppm), avdenCHECK = mean(AvDen) )  NO3Summary <- data.frame(NO3Summary)  NO3idx <- apply(fulldata, 1, function(x) { idx <- which( NO3Summary$YR == x[1] & NO3Summary$Field == x[2])  ret <- NA  if(length(idx) >0 ) ret <- NO3Summary[idx,3]  ret} )  fulldata$NO3 <- NO3idx  # NO3  model1 <- lm( log(AvDen + 1 ) ~ YR * NO3, data = fulldata )  anova(model1) | Analysis of Variance Table  Response: log(AvDen + 1)  Df Sum Sq Mean Sq F value Pr(>F)  YR 1 0.0690 0.069027 0.2934 0.5898  NO3 1 0.0236 0.023648 0.1005 0.7522  YR:NO3 1 0.0450 0.044971 0.1911 0.6634  Residuals 69 16.2356 0.235299  > summary(model10) |
| Log Striga density V Year * Mean other weed cover | LM2 | AD_1<-read.csv("MWC_AVDEN.CSV")  # Striga Density v Mean other Weed Cover for both years .  Lm2 <- lm( AvDen ~ Mean_WC*Year, data = AD_1, )  anova(lm2)  summary(lm2) | Analysis of Variance Table  Response: AvDen  Df Sum Sq Mean Sq F value Pr(>F)  Year 1 4.452 4.4522 5.6855 0.01766 *  Mean_WC 1 1.145 1.1450 1.4622 0.22742  Year:Mean_WC 1 0.080 0.0798 0.1019 0.74976  Residuals 337 263.896 0.7831  ---  Signif. codes:  0 ‘***’ 0.001 ‘**’ 0.01 ‘*’ 0.05 ‘.’ 0.1 ‘ ’ 1 |
| Log Striga density V Year * Rice Variety | LM3 | riceData <- fulldata[which(fulldata$R_M_O == "Rice"),]  model3 <- lm( log( AvDen + 1) ~ YR * CVclean , data = riceData )  anova(model3) | Analysis of Variance Table  Response: log(AvDen + 1)  Df Sum Sq Mean Sq F value Pr(>F)  YR 1 0.0989 0.09886 0.5655 0.453129  CVclean 27 9.5141 0.35237 2.0157 0.004041 **  YR:CVclean 9 2.9965 0.33294 1.9045 0.054556 .  Residuals 164 28.6697 0.17482  ---  Signif. codes:  0 ‘***’ 0.001 ‘**’ 0.01 ‘*’ 0.05 ‘.’ 0.1 ‘ ’ 1 |
| Log Striga density V Year * Previous crop | LM4 | Model4 <- lm(log( AvDen + 1) ~ YR * PC, data = fulldata)  anova(model4) | Analysis of Variance Table  Response: log(AvDen + 1)  Df Sum Sq Mean Sq F value Pr(>F)  YR 1 0.595 0.59460 3.2503 0.07268 .  PC 23 4.314 0.18757 1.0253 0.43411  YR:PC 6 2.425 0.40415 2.2092 0.04293 *  Residuals 238 43.540 0.18294  ---  Signif. codes:  0 ‘***’ 0.001 ‘**’ 0.01 ‘*’ 0.05 ‘.’ 0.1 ‘ ’ 1 ---  R-sq.(adj) = 0.123 Deviance explained = 19.5%  GCV = 263.8 Scale est. = 241.21 n = 225  > |
| Log Striga density V Year * Previous crop Legume | LM5 | Model5 <- lm(log( AvDen + 1) ~ YR * PCL, data = fulldata)  anova(model5) | Analysis of Variance Table  Response: log(AvDen + 1)  Df Sum Sq Mean Sq F value Pr(>F)  YR 1 0.809 0.80850 4.3286 0.03828 *  PCL 1 1.194 1.19366 6.3907 0.01196 *  YR:PCL 1 0.004 0.00389 0.0209 0.88528  Residuals 316 59.023 0.18678  ---  Signif. codes:  0 ‘***’ 0.001 ‘**’ 0.01 ‘*’ 0.05 ‘.’ 0.1 ‘ ’ 1 |
| Log Striga density V Year * intercrop | LM6 | Model6 <- lm(log( AvDen + 1) ~ YR * CC, data = fulldata)  anova(model6) | Analysis of Variance Table  Response: log(AvDen + 1)  Df Sum Sq Mean Sq F value Pr(>F)  YR 1 2.026 2.02550 11.5209 0.0008233 ***  CC 25 4.950 0.19801 1.1262 0.3153817  YR:CC 6 0.507 0.08446 0.4804 0.8225375  Residuals 209 36.744 0.17581  ---  Signif. codes:  0 ‘***’ 0.001 ‘**’ 0.01 ‘*’ 0.05 ‘.’ 0.1 ‘ ’ 1 |
| Log Striga density V Year * Neighboring Striga density | LM7 | # Functions to find neighbour densities  findNN <- function( p, pts) {  dists <- distm(p, pts, fun = distHaversine )  idxN1 <- which( dists == min( dists ) )  idxN2 <- which( dists == min( dists[-idxN1] ) )  idxN3 <- which( dists == min( dists[-c(idxN1, idxN2) ] ) )  return( c(idxN2, idxN3) )  }  NNdens <- function(pts, dens) {  idxs <- t( apply( pts, 1, function(x) findNN(x, pts)) )  Ns <- apply(idxs,1, function(idx) mean(dens[idx]) )  return(Ns)  }  # Run  data2019 <- fulldata[ which(fulldata$YR == 2019), ]  data2020 <- fulldata[ which(fulldata$YR == 2020), ]  ptest <- c( data2019$Lon[1], data2019$Lat[1] )  ptstest <- cbind(data2019$Lon , data2019$Lat )  Neigh2019 <- unlist( NNdens( ptstest, data2019$AvDen ) )  ptest <- c( data2020$Lon[1], data2020$Lat[1] )  ptstest <- cbind(data2020$Lon , data2020$Lat )  Neigh2020 <- unlist( NNdens( ptstest, data2020$AvDen ) )  fulldata$Neigh <- c( Neigh2019, Neigh2020 ) model7 <- lm( log( AvDen + 1) ~ YR * Neigh, data = fulldata)  anova(model7) | Analysis of Variance Table  Response: log(AvDen + 1)  Df Sum Sq Mean Sq F value Pr(>F)  YR 1 0.582 0.58238 3.0426 0.08202 .  Neigh 1 1.115 1.11534 5.8270 0.01631 *  YR:Neigh 1 1.211 1.21054 6.3244 0.01237 *  Residuals 338 64.697 0.19141  ---  Signif. codes:  0 ‘***’ 0.001 ‘**’ 0.01 ‘*’ 0.05 ‘.’ 0.1 ‘ ’ 1 |
| Log Striga density V Year * Mean annual rainfall | LM8 | Model8 <- lm( log(AvDen + 1) ~ YR * MeanRF, data = fulldata )  anova(model8) | Analysis of Variance Table  Response: log(AvDen + 1)  Df Sum Sq Mean Sq F value Pr(>F)  YR 1 1.159 1.15903 5.9334 0.015281 *  MeanRF 1 0.360 0.36001 1.8430 0.175344  YR:MeanRF 1 2.793 2.79326 14.2994 0.000179 ***  Residuals 411 80.285 0.19534  ---  Signif. codes:  0 ‘***’ 0.001 ‘**’ 0.01 ‘*’ 0.05 ‘.’ 0.1 ‘ ’ 1 |
| Log Striga density V Year * Precipitation seasonality | LM9 | Model9 <- lm( log(AvDen + 1) ~ YR * RFCV_MAN, data = fulldata )  anova(model9) | Analysis of Variance Table  Response: log(AvDen + 1)  Df Sum Sq Mean Sq F value Pr(>F)  YR 1 1.159 1.15903 5.8746 0.015791 *  RFCV_MAN 1 1.732 1.73222 8.7799 0.003223 **  YR:RFCV_MAN 1 0.618 0.61849 3.1349 0.077375 .  Residuals 411 81.088 0.19729  ---  Signif. codes:  0 ‘***’ 0.001 ‘**’ 0.01 ‘*’ 0.05 ‘.’ 0.1 ‘ ’ 1  > |
| Log Striga density V Year * Altitude | LM9 | Model10 <- lm( log( AvDen + 1 ) ~ YR * Alt, data = fulldata)  anova(model10) | Analysis of Variance Table  Response: log(AvDen + 1)  Df Sum Sq Mean Sq F value Pr(>F)  YR 1 1.103 1.10319 5.5557 0.018891 *  Alt 1 1.827 1.82653 9.1985 0.002576 **  YR:Alt 1 0.100 0.10029 0.5051 0.477691  Residuals 409 81.215 0.19857  ---  Signif. codes:  0 ‘***’ 0.001 ‘**’ 0.01 ‘*’ 0.05 ‘.’ 0.1 ‘ ’ 1 |
| Log Striga density V Year * Mean annual temperature | LM11 | Model11 <- lm( log(AvDen + 1) ~ YR * MeanTA, data = fulldata )  anova(model1) | Analysis of Variance Table  Response: log(AvDen + 1)  Df Sum Sq Mean Sq F value Pr(>F)  YR 1 1.159 1.15903 5.8923 0.0156356 *  MeanTA 1 2.481 2.48108 12.6135 0.0004273 ***  YR:MeanTA 1 0.113 0.11345 0.5768 0.4480112  Residuals 411 80.844 0.19670  ---  Signif. codes:  0 ‘***’ 0.001 ‘**’ 0.01 ‘*’ 0.05 ‘.’ 0.1 ‘ ’ 1 |
| Log Striga density V Year * Legume Crop | lm12 | LC_1<-read.csv("LEGUME_CROP_2019_2020_SINGLE_RECS_REMOVED.CSV")  # (2) Look at effects of different legume crops  # Set contrasts so that we can test against the grand mean.  options(contrasts = c("contr.sum","contr.poly"))  lm1 <- lm( log( AvDen + 1 ) ~ YR * LC, data = LC_1)  anova(lm12)  summary(lm12) | Analysis of Variance Table  Response: log(AvDen + 1)  Df Sum Sq Mean Sq F value Pr(>F)  YR 1 1.4086 1.40863 8.6945 0.003772 **  LC 6 1.7735 0.29558 1.8244 0.098934 .  YR:LC 3 1.1541 0.38469 2.3744 0.073025 .  Residuals 133 21.5479 0.16201  ---  Signif. codes:  0 ‘***’ 0.001 ‘**’ 0.01 ‘*’ 0.05 ‘.’ 0.1 ‘ ’ 1 |
| Linear model to obtain weighting coefficients for individual management components | Lm13 | AD_1<-read.csv("CD_1_With_Score.CSV")  # Density change v Total Score (Fallow, Cereal years, legume years,Numer of crops). lm13 <- lm( Change ~ FL_YR + CR_YR + LM_YR + NC, data = AD_1, )  anova(lm1) | Analysis of Variance Table  Response: Change  Df Sum Sq Mean Sq F value Pr(>F)  FL_YR 1 0.153 0.1528 0.1196 0.73048  CR_YR 1 0.055 0.0553 0.0433 0.83572  LM_YR 1 8.416 8.4164 6.5880 0.01232 *  NC 1 2.498 2.4983 1.9555 0.16623  Residuals 73 93.261 1.2775 |
| Change in average Striga density (2019-2020) v Management score |  | AD_1<-read.csv("CD_1_With_Score_Using_Coefficients.CSV")  # Average Density 2020 v Total Score (Fallow, Cereal years, legume years,Numer of crops).  lm1 <- lm( Change ~ Total, data = AD_1, )  anova(lm1) | Analysis of Variance Table  Response: Change  Df Sum Sq Mean Sq F value Pr(>F)  Total 1 11.123 11.1228 9.0642 0.003537 **  Residuals 76 93.261 1.2271  ---  Signif. code |
