# Supplementary figures and images for: Identifying existing management practices in the control of Striga asiatica within rice–maize systems in mid‐west Madagascar
Source: Ecol Evol. 2021 Sep 12;11(19):13579–92. doi: 10.1002/ece3.8085 (PMC8495792; doi:10.1002/ece3.8085)

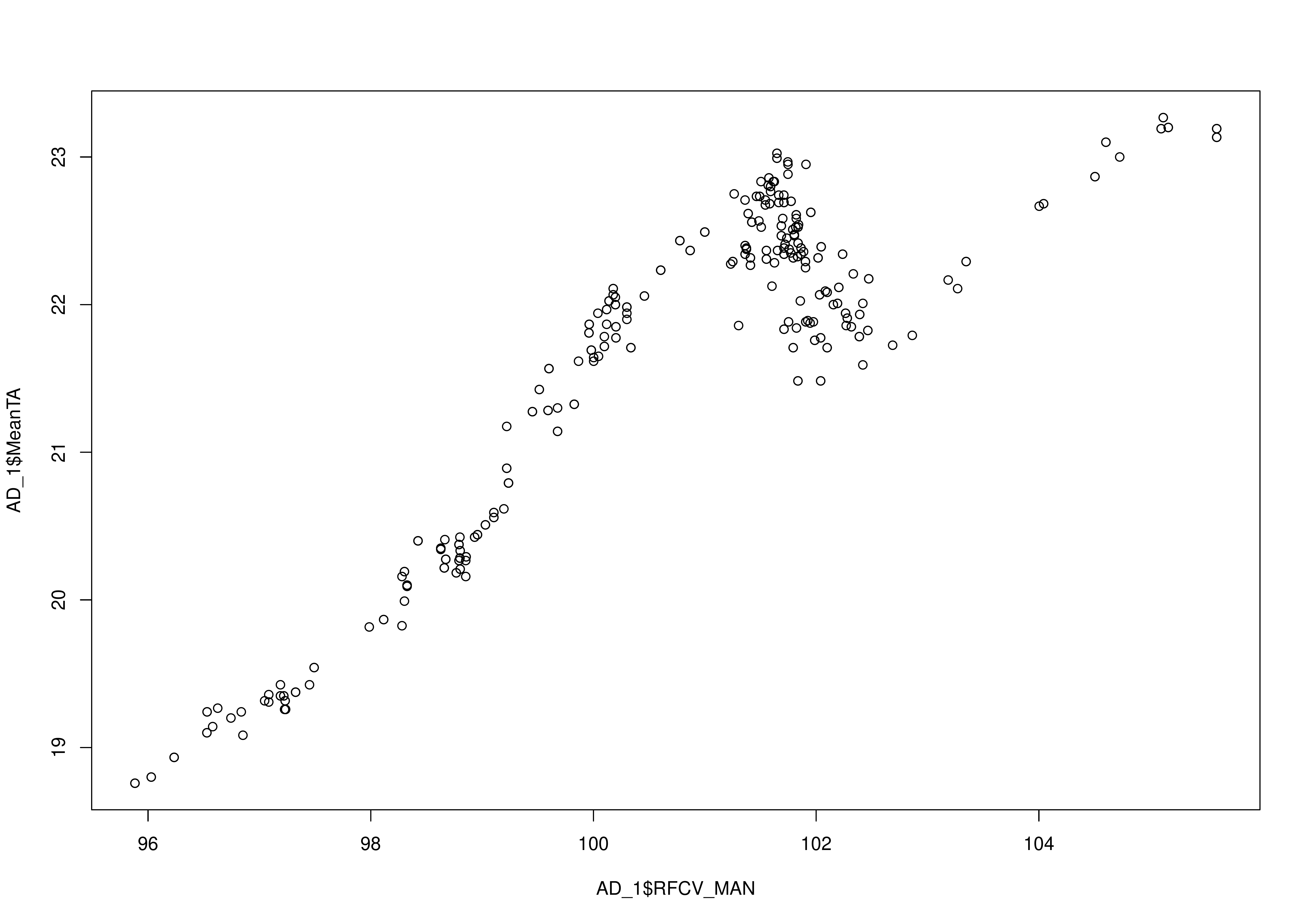

Supplement: Supplementary file 3 — Appendix S3 [file ECE3-11-13579-s002.jpg]
